# On-the-Job Safety During Enlarging an Intensive Care Unit for the COVID-19 Pandemic: Team-Based Approach with Low Infection Rate of the Staff

**DOI:** 10.5152/TJAR.2021.21116

**Published:** 2022-04-01

**Authors:** Simon Rauch, Ivo Beat Regli, Berenice Martínez Salazar, Paolo Mario Seraglio, Matteo Zanovello, Guido Schüpfer, Matthias Bock

**Affiliations:** 1 Department of Anaesthesia and Intensive Care, “F. Tappeiner” Hospital, Merano, Italy; 2 Institute of Mountain Emergency Medicine, Eurac Research, Bolzano, Italy; 3 Independent Researcher, Ora, Italy; 4 Technical Department, “F. Tappeiner” Hospital, Merano, Italy; 5 Department of Anaesthesia, Cantonal Hospital, Lucerne, Switzerland; 6 Department of Anaesthesia, Perioperative Medicine and Intensive Care, Paracelsus Medical University, Salzburg, Austria

**Keywords:** Coronavirus, infection, intensive care, leadership, management, teamwork

## Abstract

**Objective:**

Healthcare workers had a 7.4-fold risk of severe coronavirus disease-19 than non-essential employees in the United Kingdom during the first phase of the pandemic. In this study, we describe interdisciplinary measures for increasing on-the-job safety used during the first phase of the pandemic in an Italian hospital.

**Methods:**

We converted an intensive care/intermediate care unit into a fully equipped 16-bed intensive care unit with adjustments for infection control and on-the-job safety within 4 days. We compared our actions with a recently published concept on team management in the pandemic and described the implementation of each issue. It was our principal goal in this completely unknown emergency to guarantee safety for both staff and patients. We defined independent pathways for staff, patients, material, and waste. Clear procedures were defined for protecting the employees and for creating a working environment that minimizes mistakes despite challenging conditions.

**Results:**

From March 7 to April 29, we treated 34 mechanically ventilated patients in our intensive care unit with a mean bed occupancy rate of 62%. The team worked in the upgraded intensive care unit with an increased perception of safety. After cessation of the first wave of the pandemic, we tested the department’s entire staff for antibodies against severe acute respiratory syndrome coronavirus 2. Totally 2 of 122 (1.6%) team members developed anti-severe acute respiratory syndrome coronavirus 2 immunoglobulin-G antibodies during the intensive care unit’s running time.

**Conclusion:**

The successful implementation of theoretical concepts on team management into clinical practice was crucial for staff safety and on-the-job safety during the pandemic.

**Main Points**

- We describe the implementation of a bundle of measures during the enlargement and the preparation of an intensive care unit for the pandemic.
- Two of 122 (1.6%) team members had positive serology for anti-severe acute respiratory syndrome coronavirus 2 immunoglobulin-G in May 2020.
- Our team-based approach leads, therefore, to the increased on-the-job safety during a previously unknown condition.

## Introduction

During the first phase of the pandemic caused by the severe acute respiratory syndrome coronavirus 2 (SARS-CoV-2), healthcare providers had a 7.4- to 11.6-fold increased risk of severe infection in the UK and US.^1^ This underlines the importance of on-the-job safety while treating patients affected by coronavirus disease-19 (COVID-19).

On March 6, 2020, a local task force decided that the intensive care unit (ICU) of the “F. Tappeiner” hospital in Merano, province of South Tyrol, Italy, had to treat exclusively COVID-19 patients. We, therefore, enlarged our ICU capacity and upgraded it for infectious patients. We evacuated all the patients on March 7, just before the first patient affected by respiratory failure due to SARS-CoV-2 infection arrived. On Monday, March 9, we elaborated a strategy to enlarge the ICU, in strict collaboration with the hospital management and the hospital’s technical department. On March 11, we finished the operations on enlarging the ICU. Tannenbaum et al.^2^ recently proposed a bundle of 7 evidence-based measures to improve teamwork during the crisis (Table 1). Here, we describe the implementation of the concepts mentioned above in our ICU and report our staff’s positive serology rate.

At the beginning of the pandemic, there were the following significant challenges: facing a largely unknown infectious disease with a rapidly growing number of critically ill patients, transferring patients not affected by SARS-Cov-2 from our ICU to other facilities, and treating contemporaneously emergent, obstetric, and oncologic cases. Moreover, we had to cope with fear and distress among the collaborators who were concerned about their personal safety, their families’ safety, and who were worried about their physical ability to deal with the workload and the psychological stress of this crisis.^3-5^ Thus, several adjustments in staff, infrastructure, equipment, and organization were necessary. It was crucial to adapt our procedures in such a way that we could minimize the SARS-CoV-2 infection risk for our staff while offering our patients the best possible medical care.

## Methods

We categorized our measures according to the measures raised by Tannenbaum and co-workers in brackets “{}”.

### Biosafety Considerations

Severe acute respiratory syndrome coronavirus 2 was classified biosafety level 3 of 4 hazard levels (pathogens causing severe and potentially lethal diseases that aerosols can transmit).^6,7^ Consequently, we defined contaminated and clean areas and shut down elective surgical procedures on March 9. Operating room nurses and members of the hygiene department developed training programs for donning and doffing of the mandatory personal protective equipment (PPE). As a first step, all the collaborators working in the ICU and departments treating COVID-19 patients (physicians, nurses, and non-medical collaborators) were instructed by training sessions, hand-outs, and video clips produced by the hygiene department {3 and 5}. The remaining departments of the hospital were trained in the second step. The participants then repeated the sessions to their local teams. Donning and doffing the PPE were performed in teams to increase personal safety {3 and 5}.

We further emphasized the importance of hand hygiene using the World Health Organization recommendations as a basis.^8^ Although SARS-CoV-2 survives several hours in aerosols and several days on various surfaces,^9,10^ it is highly susceptible to alcohol-based disinfectants.^11,12^ Therefore, both equipment and medical supplies had to be easy to clean and decontaminate. 

We further reduced ambient air pressure in the patient rooms in comparison to the rest of the ICU by increasing air aspiration in the single-patient rooms. Whenever possible, we performed bedside diagnostics and procedures in order to avoid the patient’s contact outside the ICU. Even in an end-of-life situation, the strict interdiction of relatives’ visits was a further action for infection control during the first phase of the pandemic. We invited all the relatives to contact the ICU staff at any time to compensate for the lack of physical contact with the patients. The patients themselves received tablets for communication with their relatives as soon as they could handle the devices. One physician contacted the relatives daily to inform them about the clinical status of the patient. This had a positive side-effect for the team members; one physician had the opportunity to work without PPE as this was done outside of the contaminated ICU area.

### Team Approach for Reorganization

We informed the team immediately about the primary goal of all the measures: to guarantee safety for both staff and patients. Then, everybody was involved in designing the adjustments of infrastructure and workflow of the ICU.

We adapted both infrastructure and staff management to the COVID-19 operation mode in a way that minimizes both active and latent failures, the most significant reasons for adverse events.^13,14^ Table 2 shows the workplace-related measures elaborated according to previously published design guidelines.^15,16^

We operated in the COVID-19 ICU during a period where research produced an impressive amount of data regarding the characteristics and possible treatments of the new disease. Therefore, we aimed to provide easily accessible information by gathering current protocols and literature on a central data server and exchanging crucial information on a phone-based instant messaging application. 

We further emphasized the value of open and blame-free communication and a positive error culture, especially during the pandemic. We encouraged the team members to talk openly about workload and exhaustion {5 and 6}. All the team members received an adequate number of breaks to reduce errors due to exhaustion during the PPE shifts or doffing. These breaks were individualized for the following reasons: we extended the duration of the breaks up to an hour if possible and invited all the team members to ask for additional breaks whenever necessary. Therefore, the breaks’ timing and duration were depended on the collaborators’ physical conditions instead of a fixed schedule. These measures permitted us to organize shifts of 12 hours, thereby minimizing the number of handovers.

### Infrastructure

**Figure 1.**
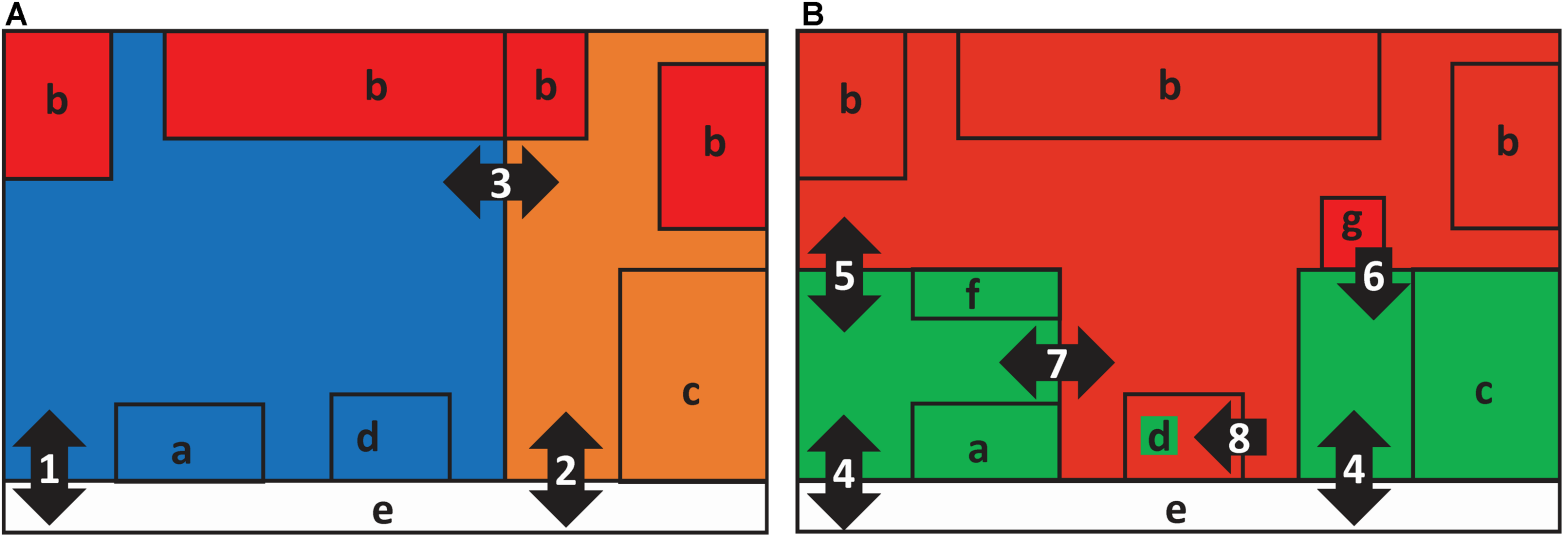
Schematics of the ICU and cardiology IMC before (A) and during (B) the first wave of the COVID-19 pandemic. (A) Blue, ICU; Orange, cardiology IMC; Red, areas with a potential for microbiological contamination; White, area outside of the ICU and IMC; (a) locker room, (b) patient rooms, (c) meeting rooms and offices, (d) material elevator, (e) hospital hallway; (1) entry and exit to the ICU, (2) entry and exit to the cardiology IMC, (3) passage between ICU and cardiology IMC. (B) Green, clean area of the COVID-19 ICU; Red, area of the COVID-19 ICU with a potential for microbiological contamination; White, area outside of the COVID-19 ICU; (a) locker room, (b) patient rooms, (c) break rooms, (d) material elevator, (e) hospital hallway, (f) changing area for personal protection equipment, (g) personal protective equipment removal area; (1) entries and exits to the (clean) COVID-19 ICU, (2) access to the contaminated COVID-19 ICU for staff/access to and exit from the contaminated COVID-19 ICU for patients, (3) exit from the contaminated COVID-19 ICU for staff, (4) supplies and sample gate to the contaminated ICU, (5) access from the contaminated ICU to the (clean) material elevator. ICU, intensive care unit; IMC, intermediate care unit; COVID-19, coronavirus disease 19.

Our hospital was equipped with a 9-bed ICU and a 7-bed intermediate care unit (IMC). During the COVID-19 pandemic, we upgraded the IMC beds to ICU beds and obtained a 16-bed COVID-19 ICU mainly composed of single-patient rooms (Figure 1A). During the first phase of the pandemic, PPE availability was limited and a single-box design increases the number of donning and doffing procedures and thereby wastage; hence, we defined the central area of the ICU as a contaminated zone. Furthermore, at that time, we hypothesized that such an ICU design might reduce the risk of infection. We separated this zone from the clean area using provisional walls made of laminated wood. All the materials for reconstruction were easy to disinfect. Staff, patients, and materials were routed separately in and out of the ICU {3 and 7}. We converted former meeting rooms in the green area into separate break rooms and lunchrooms. Additional break rooms were also created outside the ICU in the clinic for preoperative visits. Figure 1B shows the design of the ICU after the expansion to 16 beds.

Supplies, samples, and garbage could only be introduced into the contaminated zone and removed from it via dedicated material gates (Figure 1B).^17^ We stored the ICU equipment centrally within the red area and kept it always ready for use. The whole equipment was always accessible from inside the ICU. Besides, we set up a backup storage room with a stock of crucial ICU supplies next to the material gate.^17^ One team member of the nursing staff organized the restoration of ICU supplies and PPE to facilitate smooth ICU operations. This collaborator also calculated the need for PPE supply based on the fundamental shifts and breaks.

### Procedures

#### Personal Protective Equipment

We decided to restrict access to the ICU to authorized persons on a need-to-enter basis. We dressed in work clothing in a locker room before moving to the donning area to enter the contaminated area. The standard PPE in the contaminated area was composed of a face mask, protective glasses, protective coat or overall, hood, shoe covers, and 2 pairs of gloves. Visors, extra gloves, and adhesive tape were also available to protect further and seal the equipment. Before exiting, we passed a dedicated PPE equipment removal area.

#### Waste Management

We removed PPE in a specific area before leaving the contaminated zone. We placed re-usable equipment parts (visors and protective goggles) in tubs with disinfectant before shipping to the sterilization unit. Non-reusable items of equipment were discarded. We collected waste in bags of hazardous hospital waste and transported it to the clean zone by elevator. All the waste was further transferred to a certified disposal authority for burning. We managed dirty laundry by double bagging it before transporting it with an elevator to the hospital laundry for disinfection.

#### Patient Transport to the ICU

We treated exclusively patients requiring invasive ventilation for COVID-19-associated respiratory failure. Other wards re-designed as IMC units took care of the remaining patients. The physicians working in these units contacted us regularly in order to schedule a possible admission to ICU. We further discussed critical patients’ conditions within our hospital during daily meetings with the management and all other hospital departments. We continuously evaluated the patients on the regular COVID-19 wards and closely collaborated with the teams responsible for these patients. Patients admitted to the ICU entered the hospital through a dedicated hospital entrance. During this transfer, we blocked the route to the ICU for others to minimize contact with the patient. Patients transferred from another ward within our hospital to the ICU approached the elevator through an underground tunnel. These patient routes were marked with large and visible arrows on the floor. Patients leaving the contaminated zone took the same paths as patients entering the ICU (Figure 1B). 

#### Working in the Contaminated Zone

We followed the security measures crucially. The outermost pair of gloves was changed after every patient manipulation. All the maneuvers on the patients were conducted without disconnection from the ventilator. We optimized the administration times of pharmacotherapy to minimize contact between the nursing staff and the patients. Additional protective measures were taken before performing an aerosol-producing measure: limiting the number of personnel in the room, double-checking the protective gear’s correct positioning, and wearing a face shield. We used video-laryngoscopes and single-use bronchoscopes to reduce pathogen spread. After completing an aerosol-producing measure, we thoroughly cleaned and disinfected the zone where the procedure took place.

#### Diagnostic Tests

We used a test with a sensitivity of 97.9% and a specificity of 98.5% (LIAISON^®^ SARS-CoV-2 S1/S2 IgG, anti-spike IgG antibody Diasorin, Saluggia, Italy) for the screening of employees. We did not use tests on immunoglobulin (Ig)-M antibodies as we did not intend to analyze acute infection. The following real-time reverse transcription-polymerase chain reaction (RT-PCR) was used to confirm the diagnosis of SARS-Cov-2 in our patients: Xpert Xpress SARS-CoV-2 (Xpert) (Cepheid, Buccinasco, Italy), Seegene 2019-nCOV Assay RP10245X (Seegene, Genova, Italy) and a validated European diagnostic workflow.^18^

## Results

In May 2020, we tested the entire staff of 122 collaborators for anti-SARS-CoV-2 IgG antibodies. In total, 2 out of 28 (7.1%) physicians and 2 out of 68 (2.9%) nurses presented anti-SARS-CoV-2 antibodies, whereas none of the auxiliary non-medical collaborators (n = 26) was serum-positive. Three team members did not give consent for antibody testing. One collaborator had a positive serology due to an infection (confirmed by positive RT-PCR at the beginning of March 2020) that occurred before the arrival of the first patient. Further tests showed that another collaborator had a false-positive test result. Thus, 2 of 122 (1.6%) collaborators tested developed antibodies during the period they worked in the ICU. After a critical review of the circumstances of the infections and the collaborators, 1 of these infections likely occurred outside the hospital. Most likely, an incident (loss of the correct position of the mask and inadvertent disconnection of a patient from the ventilator) within the ICU induced the second infection. We did not perform regular PCR tests of asymptomatic team members during phase 1 of the pandemic.

**Figure 2.**
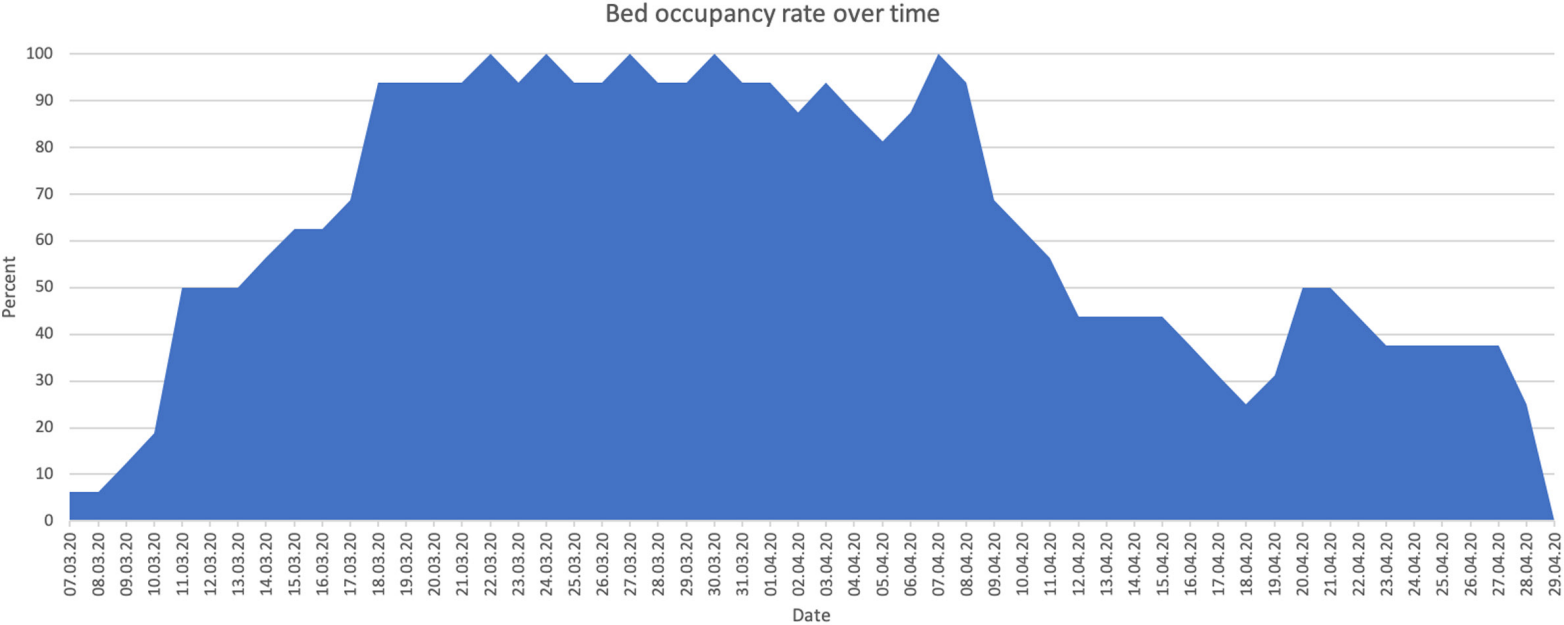
Bed occupancy rate. The graph depicts the percentage of bed occupancy of the ICU during the first wave of the pandemic. ICU, intensive care unit.

Table 3 provides clinical data of the patients treated in the ICU. We treated 4 patients with continuous venovenous haemodiafiltration. No patient required treatment with extracorporeal membrane oxygenation. The mean occupancy rate was 62% (Figure 2). On April 29, 2020, the last patient with COVID-19 was discharged from our COVID-19 ICU, and the ward was restored to its original status. Diagnosis of SARS-Cov-2 infection was confirmed by RT-PCR of nasal swaps and bronchoalveolar lavage in all patients. We did not perform antibody tests on the patients.

## Discussion

From March 7 to April 29, we took care of 34 patients. All of them required mechanical ventilation. Nine patients were deceased (26%) and the remaining 25 patients were discharged (74%). The median age was higher among deceased patients compared to survivors (71 years vs 61 years, *P* < .05). The median length of stay in the ICU was 17 days (9 days in deceased patients vs 19 days among survivors, n. s.). The median duration of invasive mechanical ventilation was 17 days (9 days for deceased patients vs 17 days among survivors, n. s.). 

The l task force coordinated the allocation of patients from other facilities in South Tyrol to our ICU and later during the pandemic, the allocation to all the ICUs of South Tyrol. Analogs to authorities in Germany and Austria, we prepared a flowchart of the indications for treatment in the ICU together with the local ethics committee and the regional task force in order to be prepared in the case of overload of the ICU capacity. This was necessary at the beginning of the pandemic in our province as the infrastructure of ICU beds was one of Italy’s most significant problems during the crisis. Italy’s largest center for significant surgery in Milan reported that 28 ICU beds were enlarged to 72 ICU beds during the pandemic.^19^

Like other studies on screening health care workers, we only performed a single test for each collaborator.^20,21^ The rate of false-positive results is significant when testing among a small population of employees. Our results with 1 false-positive test are therefore in line with this specification. Unfortunately, we cannot compare the rate of positive serology among our employees with the prevalence within the population due to the lack of data among the general population. The seropositive rate of antibodies against SARS-CoV-2 among all the staff members at our hospital was 3.1% (52 out of 1666 collaborators tested) in May 2020. A tertiary care facility in Belgium reports a seropositive rate of 6.4% among the hospital staff.^20^ The prevalence of SARS-CoV-2 IgG was 7.4% among healthcare workers in Milan, Italy, in April 2020.^21^ A population-based study in the Canton of Geneva, Switzerland, estimated a seroconversion rate in the population between 6.6% and 10.9% by the end of April/beginning of May 2020.^22^ We therefore conclude that our staff was not exposed to an increased risk of COVID-19 infection.

In June 2020, Aziz et al^23^ published a guideline on the organizational management of ICUs caring for COVID-19 patients. Retrospectively, this guideline confirmed that our adaptations were generally in line with this expert consensus. Our data suggest that proper hospital and ICU management can minimize the spread of infections during a pandemic such as SARS-CoV-2. The bundle proposed by Tannenbaum et al^2^ provides excellent help for the management of emergencies like the COVID-19 crisis. We implemented aspects before their publication. This emphasizes the importance of the bundle as we can exclude an expectant observer effect. We continued nearly all the measures described in this article during the second wave of the COVID-19 pandemic in Italy (i.e., from October 2020 until April 2021). However, due to new insights regarding the transmission dynamics of SARS-CoV-2^24^ and the easier availability of PPE, the contaminated zone was limited to the individual patient rooms. Therefore, wearing PPE was restricted to the period in the patient rooms, but wearing FFP-2 face masks were still mandatory in the entire hospital. Furthermore, we allowed 1 family member to visit the patients after thorough instruction in PPE use. 

We decided to implement negative pressure rooms within our ICU as airborne transmission mainly characterizes the infectious risk of SARS-CoV-2. Therefore, our basic approach for infection control was minimizing the number of aerosol-generating procedures and maintaining the potentially contaminated air within the single-patient rooms.^25^ Retrospectively, the Asian Critical Care Clinical Trials Group confirmed our strategy^26^ that physicians must be aware of the risks of negative pressure rooms, especially for immunocompromised patients: potentially hazardous pathogens like *Aspergillus fumigatus* might be directed into the patient rooms. Contrary to others, we did not perform regular antifungal^27^ or other antimicrobial prophylaxis on our patients. We performed weekly screening and tested for fungal infections when clinically indicated but did not observe infections by *A*.* fumigatus*. These findings underline the importance of strict hygiene concepts in the whole ICU and the air management systems.

Major limitations include the retrospective study design. We did not perform RT-PCR tests or IgM antibody screening on asymptomatic staff members. Antibody testing reflected the prevalence of a positive serology only in May 2020. We cannot rule out that staff members had clinically asymptomatic infections or did not produce the relevant antibodies.^28^ Kinetics of SARS-CoV-2 IgG, however, usually do not differ from the kinetics of antibodies after other viral infections and remain for at least 3 months.^29^ Moreover, our experience is restricted to a relatively small ICU in a single center. Our bed occupancy rate was relatively low. This emphasizes the importance of the local network like in South Tyrol as we avoided a triage situation with a negative impact on patient outcome. Moreover, the strict collaboration with the other departments within the hospital helped to treat critical patients outside the ICU.

Our structured team-based mode of action with the following main issues was successful during the challenge of the actual pandemic. The following issues were most important: we informed the collaborators and involved the employees, the hospital management, and the technical departure in the upcoming decisions. All the team members participated in the implementation of the adjustments. The measures mentioned above increased the team members’ confidence and generated a sensation of safety and trust during the first phase of the pandemic. Many collaborators even called the new ward “our” ICU. This work also underlines the importance of a bundle of measures for on-the-job safety during the pandemic. Caregivers can provide safe working conditions, but each team member should give his own active contribution by adhering to vaccination campaigns. Thus, every single professional will further increase safety for the whole team and for the patients treated by the team.

### Declaration of Interest:

The authors have no conflict of interest to declare.

## Figures and Tables

**Table 1:** Risk Points for Teams Working in the Pandemic (Mod. from^1^)


Risk Points for Team Performance
Uncertainty or doubt the success of the team
Competing or inconsistent mental models, narrowing of attention and debriefings
Manifestation of schisms, fault lines; silos Insufficient monitoring, vigilance, backup; narrowing of attention, low psychological safety Discomfort with speaking up: lack of psychological safety Narrowing of attention; overfocus on oneself; reduced vigilance Setbacks adversely affecting readiness to perform subsequent tasks; low team resilience

**Table 2.** Measures to Avoid Latent Errors and Structural Adaptations

Staff Management	Structural Adaptations
Involvement of the whole team in planning of COVID-19-specific ICU organization {2, 4}	Definition of clean and contaminated zones
Integration of the whole team in the execution of the measures {1, 2, 5}	Usage of easy to disinfect equipment only
Daily prebriefings and debriefings {1, 2, 4, 7}	Hypobaric zones in the contaminated areas and inpatient rooms
Definition and regular improvement of a treatment protocol {2}	Definition of specific entrance and exit gates for the contaminated zone for patients, staff, and material
Easily accessible and up to date information management (central data server, instant messaging via smartphone application) {2}	Establishment of routes for patient transports Bedside execution of medical procedures
Constant exchange with hospital management and technical department	
Minimizing the number of patient handovers by 12 hours shifts {5} Creation of a designated handover sheet {2, 7} Flexible break times {6, 7} Creation of additional break rooms {6, 7}	

COVID-19, coronavirus disease 2019; ICU, intensive care unit.

**Table 3.** Clinical Parameters on Patients Treated in the COVID-19 ICU

	Number	Percentage	Median Age (Range)	Median Duration of Stay (Range)	Median Duration of Mechanical Ventilation^1^ (Range)
Total	34	100	67 years (28-80 years)	17 days (1-81 days)	17 days (2-77 days)
Deceased	9	26	71 years (64-80 years)	9 days (2-61 days)	9 days (2-61 days)
Survived	25	74	61 years (28-80 years)	19 days (3-81 days)	17 (3-77 days)
*P* value^2^			.012*	.495	.214

Clinical parameters on the 34 patients treated from March 7 to April 29, 2020 in the COVID-19 ICU. ^1^Three missing datapoints. ^2^ *P*-values of comparison of deceased versus survived using the Wilcoxon–Mann–Whitney test, *P< .05. COVID-19, coronavirus disease 2019; ICU, intensive care unit.
